# Development of New Candidate Gene and EST-Based Molecular Markers for *Gossypium* Species

**DOI:** 10.1155/2011/894598

**Published:** 2012-01-17

**Authors:** Ramesh Buyyarapu, Ramesh V. Kantety, John Z. Yu, Sukumar Saha, Govind C. Sharma

**Affiliations:** ^1^Center for Molecular Biology, Department of Natural Resources and Environmental Sciences, Alabama A&M University, 134 ARC Building, P.O. Box 1927, Normal, AL 35762, USA; ^2^Southern Plains Agricultural Research Center, USDA-ARS, 2881 F&B Road, College Station, TX 77845, USA; ^3^Genetics and Precision Agriculture Research Unit, USDA-ARS, P.O. Box 5367, MS 39762, USA

## Abstract

New source of molecular markers accelerate the efforts in improving cotton fiber traits and aid in developing high-density integrated genetic maps. We developed new markers based on candidate genes and *G. arboreum* EST sequences that were used for polymorphism detection followed by genetic and physical mapping. Nineteen gene-based markers were surveyed for polymorphism detection in 26 *Gossypium* species. Cluster analysis generated a phylogenetic tree with four major sub-clusters for 23 species while three species branched out individually. CAP method enhanced the rate of polymorphism of candidate gene-based markers between *G. hirsutum* and *G. barbadense*. Two hundred A-genome based SSR markers were designed after datamining of *G. arboreum* EST sequences (Mississippi *Gossypium arboreum* EST-SSR: MGAES). Over 70% of MGAES markers successfully produced amplicons while 65 of them demonstrated polymorphism between the parents of *G. hirsutum* and *G. barbadense* RIL population and formed 14 linkage groups. Chromosomal localization of both candidate gene-based and MGAES markers was assisted by euploid and hypoaneuploid CS-B analysis. Gene-based and MGAES markers were highly informative as they were designed from candidate genes and fiber transcriptome with a potential to be integrated into the existing cotton genetic and physical maps.

## 1. Introduction

Molecular markers provide valuable information in assessing the genetic variability, generating linkage maps, enabling better understanding of the genome organization, and deciphering quantitative trait loci (QTLs). Initial effort to map the cotton genome using an F_2_ population utilized 705 restriction fragment length polymorphism (RFLP) probes that were polymorphic between *G. hirsutum* and *G. barbadense* and generated 41 linkage groups spanning 4675 cM [1]. Genetic variation at molecular level in cotton has been characterized using isozyme/allozyme markers [2], RFLPs [1, 3, 4], AFLPs [5, 6], and microsatellites [7, 8] in *G. hirsutum* and its related species. A comprehensive comparative genetic map with 2584 loci at *∼*1.72 cM intervals for tetraploid (A_t_D_t_) cotton and with 662 loci at *∼*1.96 cM intervals for diploid (D) genome was constructed using RFLPs, genomic SSRs, and sequence tagged sites (STS) as probes [9]. 

Advances in technology have facilitated sequencing of complete transcriptomes and genomes that are accessible through public domain databases. Increasing number of expressed sequence tags (ESTs) for cotton facilitated the identification of simple sequence repeat (SSR) regions from the ESTs through data mining techniques. EST-SSR markers reveal putative functional genes and aid in map-based cloning of important genes [10, 11]. In cotton, several EST-SSRs have been recently mapped [12–15]. Cotton fiber genes were mapped with EST-derived SSR loci using recombinant inbred line (RIL) population derived from an interspecific cross between *G. hirsutum* × *G. barbadense *[16]. Other alternative mapping approaches such as whole-genome radiation hybrid (WGRH) mapping and fluorescent in situ hybridization (FISH) mapping have been utilized to generate an integrated cotton genome map [17].

Assignment of linkage groups or markers to chromosomes is made by use of aneuploid chromosome substitution (F_1_ Stocks) lines of *G. barbadense* in *G. hirsutum*, euploid chromosome substitution lines of *G. barbadense* in *G. hirsutum*, and aneuploid chromosome substitution (F_1_ Stocks) lines of *G. tomentosum* in *G. hirsutum *[18]. Euploid chromosome substitution stocks were developed by inbreeding the hemizygous monosomic and monotelodisomic substitution stocks through backcrossing up to BC_5_ generation [19]. Using these available substitution lines, various molecular markers, linkage groups, and QTLs for agronomic and fiber quality traits were physically mapped to different cotton chromosomes [1, 9, 20].

Current integrated genetic maps in cotton utilized mainly RFLP, AFLP, genomic SSR, STS, and EST-SSRs. However, increasing the marker density with functionally expressed genes would make the linkage maps more valuable for crop improvement programs. New sources of molecular markers such as cleaved amplified fragment polymorphism (CAP), EST-SSR, and single nucleotide polymorphism (SNP) markers expand the current but limited repertoire of existing molecular markers. In this study, our objective was to understand the genetic diversity and phylogeny of the cultivated tetraploid, and wild diploid cotton species including the Cotton Marker Database (CMD) panel through the evaluation of candidate genes, CAP and EST-SSR marker technologies, and to use these markers in the construction of integrated genetic and physical maps in cotton.

Sequence information of candidate genes available in related species for functional genes helps in designing primers to amplify and differentiate between the species. CAP markers are extensively used in human and animal sciences while they were not exploited well in plant sciences for mapping [21]. CAP is an effective technology that uses PCR and restriction digestion to elucidate the polymorphism at nucleotide level without the knowledge of the sequence information of a marker. Using the abundant sequence information available for *G. arboreum* (diploid) fiber ESTs in GenBank, we designed over 700 nonredundant primer pairs based on the identification of the SSR regions, and tested 200 primer pairs using the two major cultivated tetraploid species *G. hirsutum* (TM-1) and *G. barbadense* (3–79), the parents for RIL population used in this study. Polymorphic markers were used for genetic mapping using an RIL population, and were further physically localized through the use of monosomic, monotelosomic aneuploid, and euploid chromosome substitution lines of *G. barbadense* in *G. hirsutum* genetic background.

## 2. Materials and Methods

### 2.1. Genetic Materials

Leaf samples were collected from the Cotton Germplasm Research Unit greenhouses at USDA-ARS, College Station, TX, for (1) Cotton Marker Database (CMD) panel (12 genotypes of six *Gossypium* species) [22] and (2) other geographically diverse tetraploid species and wild diploid accessions (Table 1). Leaf samples were also collected from USDA-ARS Mississippi State, MS, for aneuploid chromosome substitution lines (BC_0_F_1_) of *G. barbadense* in *G. hirsutum* and seventeen euploid chromosome substitution lines of *G. barbadense* in *G. hirsutum*. Genomic DNA of 186 individual RILs derived from a cross between TM-1 x 3–79 were provided by USDA-ARS, Southern Plains Agricultural Research Center, Crop Germplasm Research Unit, College Station, TX.

### 2.2. DNA Extraction

Young leaf tissues were lyophilized and the DNA was extracted using the Yu lab method at USDA-ARS, College Station, TX [23]. DNA quality was evaluated using 0.7% agarose gel electrophoresis at 40 V for 3 hours. Genomic DNA was also quantified using TKO 100 fluorometer and further diluted to a working concentration of 50 ng/*μ*L for use in polymerase chain reaction (PCR).

### 2.3. Gene-Based Markers

*G. arboreum* EST sequences in GenBank were compared with non-redundant (nr) protein database to derive the putative gene function using BLASTX program. ESTs that had significant homology with functional genes in *Arabidopsis*, *Oryza*, and others were selected for polymorphism screening. Forty-seven primer pairs were synthesized (Sigma Genosys, The Woodlands, TX) based on these candidate genes that have functional significance in cotton (See Supplementary File-1 in supplementary material available on line at doi:10.1155/2011/894598). Primers were evaluated for amplification using PCR at two annealing conditions (T*_m_* = 50°C and 60–55 touchdown). Amplified products were surveyed for polymorphism using 6% polyacrylamide gel electrophoresis (PAGE) and scored in binary fashion for each fragment size. The data was used to calculate the Polymorphism Information Content (PIC) value. Cluster analysis was conducted with nearest neighborhood joining method in classifying the binary data derived to generate phylogenetic tree to assess the evolutionary relationships [24] among the five tetraploid and 21 diploid species. PCR products of the monomorphic markers between *G. hirsutum* and *G. barbadense* were subjected to digestion using *Rsa*I, *Msp*I, *Hha*I, and *Hae*III restriction endonucleases and surveyed for polymorphism using PAGE for detection of CAP markers. Fragment-based and CAP-based markers were subsequently tested for chromosomal localization in aneuploid and euploid chromosome substitution lines. 

### 2.4. EST-SSR-Based Markers

*G. arboreum* ESTs (38,893) were collected from GenBank and were searched for the presence of SSR sequences, followed by masking. The masked ESTs were clustered using StackPack v2.1 (Electric Genetics, Reston, VA) software to reduce the redundancy. The non-redundant (NR) sequences that contain an SSR motif were selected for further analysis as described by Kantety et al. [25]. A subset of NR-ESTs mainly expressed in fibers (725) were identified with having a repeat length 18 or more. Among this subset SSR containing NR-ESTs, we designed 200 primer-pairs for further analysis. They were designated as Mississippi *Gossypium arboreum* EST-SSRs (MGAES). The design of the primers was based on the sequence information flanking the SSR region with an estimated product size of ~200–300 base pairs using Primer3 software [26] and were synthesized at Sigma-Genosys (Sigma-Aldrich, Saint Louis, MO). The primer sequences, EST sources, and their putative function were summarized in Supplementary File-2. The MGAES primers were verified against all the SSR marker primer sequences available at Cotton Microsatellite Database (CMD, http://www.cottonmarker.org/) for redundancy and sequence homology using BLAST search. These MGAES and gene-based primer sequences will be submitted to CMD for cotton research community use. MGAES primers were first amplified on the RIL parents:* G. hirsutum* and *G. barbadense*, at annealing conditions of 50°C and 55°C; and surveyed for fragment length polymorphism using 6% PAGE. Polymorphic markers were then identified to genotype the RIL population for construction of genetic linkage groups. The amplified markers were also used for physical mapping onto chromosomes and chromosome arms.

### 2.5. Data Analysis

Polymorphic data was scored as binary values (1 as presence of fragment, 0 as absence) and used for the calculation of PIC value [27]. Binary data was also used to generate a phylogram using cluster analysis with SAS v9.1 (SAS Inc, Cary, NC). Similarly, binary data from RIL population for polymorphic fragments were used to create linkage groups using MapManager QTX software [28]. Recombination frequencies were converted into linkage distances using Haldane function [29]. The maximum linkage distance below 50 cM between any two markers and an LOD (logarithm of odds) score of 4 and above were considered optimal to qualify as a linkage group.

## 3. Results and Discussion

### 3.1. Candidate Gene-Based Markers

The thrust of this effort was to expand the very limited base of markers that are utilized in characterizing genetic variability in *Gossypium*. Comparative genetic approaches have been proved successful for characterizing genomes and mapping important traits based on the sequence information in related plant species [30]. Candidate genes in *G. arboreum* EST sequences were identified by comparison with distant species using BLASTX to reveal the gene function information and such ESTs were used to design primers. Nineteen candidate gene markers (40%) were successfully amplified out of 47 gene-based markers screened across the 32 genotypes from 26 diverse *Gossypium* species tested in this study. PAGE fragment analysis for amplified products identified 13 markers that were polymorphic among these cotton species (68%). Binary fragment data for these markers were used to calculate PIC value for each marker that ranged from 0.794 to 0.998 (Table 2).

Though these gene-based markers were highly polymorphic across multiple cotton species, the fragment polymorphism rates detected using direct amplicons were very low for the two cultivated tetraploids versus *G. hirsutum* and *G. barbadense.* Only one polymorphic marker was identified between *G. hirsutum* TM-1 (CMD-1) and *G. barbadense* PIMA 3–79 (CMD-2). This limitation led us to explore additional avenues by restriction digestion of the large PCR fragments to survey for polymorphism at a higher resolution. CAP markers have been used in marker-assisted selection process and mapping genetic loci of interest [21, 31]. Large amplicon sizes for many gene-based markers provided an opportunity to employ CAP technique to detect nucleotide level polymorphism for TM-1 and PIMA 3–79. To enable higher restriction site choices in these amplicons, we used *Rsa*I, *Msp*I, *Hha*I, and *Hae*III enzymes that detect four base restriction site recognition motifs. CAP technique identified eleven polymorphic markers (58%) of the 19 tested on TM-1 and PIMA 3–79 suggesting the potential for CAP technology as a useful resource for identifying genetic variation. One fragment-based and five CAP-based markers were localized to cotton chromosome or chromosome-arm using the euploid CS-B lines. Our results suggest that CAP-based marker technology is a robust approach for detection of variation in closely related species and provides an alternative to cost-intensive SNP-based approaches.

Euploid CS-B lines were annotated on the basis of the chromosome pair substituted for the complete chromosomes or chromosome arms of *G. hirsutum* monosomic or monotelodisomics [19, 32]. If a polymorphic marker between *G. hirsutum* and *G. barbadense* showed similar fragment patterns to that of *G. barbadense* in a euploid CS-B line, then that marker was concluded to be localized to particular substituted chromosome or arm. In this manner, both dominant and recessive alleles were physically mapped using euploid CS-B lines. One amplicon length polymorphism and five CAP-based markers were localized to seven chromosomes or arms using the euploid CS-B lines (Table 2). As these markers were based on homologous gene sequences, there is a possibility of having multiple copies in the tetraploid cotton genomes. Therefore this study adds a new set of gene-based markers with their specific chromosomal location and help in assessing the evolutionary relationships among the 26 *Gossypium* species.

### 3.2. Phylogenetic Analysis

*Gossypium* genus includes five tetraploid species from AD_1_–AD_5_ genomes and approximately forty-five diploid species from genome groups A–G and K [33]. Of these all five tetraploid species and twenty-one diploid species belonging to A(1), B(1), C(4), D(11), E(3), and G(1) genomes were included in this study. Relationships among these cotton genome groups were studied earlier using polymorphisms exhibited in chloroplast genome [34], ribosomal genes [35], and *Adh* genes [36]. These studies showed close relationships among the species belonging to the same geographical origin [37] besides explaining the origin of New World tetraploids from the Old World diploids [38].

**Figure 1:**
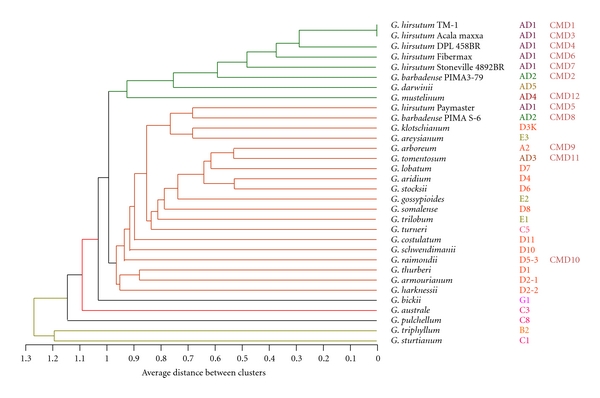
Phylogenetic relationships among the 32 *Gossypium* genotypes (Table 1) based on the fragment size polymorphism.

In this study, a total of 76 fragments from nineteen candidate gene-based markers were observed across the diverse panel of 32 cotton genotypes. Binary data derived from the fragment analysis was used to generate a phylogenetic tree providing the evolutionary relationships among the 26 cotton species by cluster analysis using maximum likelihood method (Figure 1). Cluster analysis resulted in a dendrogram comprising four major clusters grouping 23 species except that *G. bickii *(G1),* G. pulchellum* (C3), and* G. australe* (C8) branched out individually. As the dendrogram was derived based on the maximum likelihood method, it provides the evolutionary relationships based on combined genetic lineages of the candidate genes used in this study. Many genotypes from tetraploid species formed into two clades while the diploid species form the remaining. Species belonging to the same genome are grouped together to form subclusters in the dendrogram and was evident in grouping of tetraploids as wells as other diploid species. Though the number of genes used in this study is not an exhaustive data set, the evolutionary relationships among the 26 species were mostly congruent with earlier studies [37]. The dendrogram also supported the theory that *G. darwinii* (AD_5_), another tetraploid species endemic to Galapagos Islands is closely related to *G. barbadense* (AD_2_) [39, 40]. 

### 3.3. *G. arboretum* EST-SSR Markers

Availability of abundant EST information in public databases and detection procedures to identify SSR regions in ESTs have established EST-SSRs as an informative resource for genetic mapping [7, 12, 14, 25, 41]. Despite the earlier efforts by Han et al. [15] and Part et al. [16] to characterize cotton genome using EST-SSRs, the molecular variation in the coding regions of many fiber expressed genes was not yet fully utilized to assist the marker-assisted selection of important fiber traits. Two hundred primer pairs were designed specifically from fiber related ESTs were used for polymorphism detection and mapping in this study. Except 27 individual primer sequences, the rest of the MGAES markers were new and nonrepetitive based on BLAST homology search from earlier studies of Han et al. [15] and Park et al. [41]. Though these EST-SSRs were derived from a diploid progenitor (genome A_1-2_) of tetraploid species (A_t_D_t_), 147 markers (74%) out of 200 primer pairs were successfully amplified in tetraploid cultivars *G. hirsutum* TM-1 and *G. barbadense* PIMA 3–79 suggesting considerable homology exists of the tetraploid cotton with the diploid ancestral species. Sixty-five MGAES markers (44%) were polymorphic between TM-1 and PIMA 3–79 indicating the merit of these markers due to high rate of polymorphism compared to earlier studies [15]. Though these *G. arboreum* EST-SSR markers were highly polymorphic between the *G. hirsutum* and *G. barbadense* species, we observed very low polymorphism rate within each species. High polymorphic rate between the species could also be attributed to amplification of genotypes under stringent PCR conditions to avoid non-specific amplification. High levels of polymorphism were detected using *G. arboreum*-based EST-SSRs suggested the potential of cross-species transferability of these markers among diploid and tetraploid species [42]. Many of these MGAES markers were derived from the fiber expressed ESTs thus making them more valuable in breeding programs and marker-assisted selection for fiber-associated traits. Polymorphism detected for each fragment in 186 RILs was scored initially in ternary fashion and then converted into binary fashion by treating the heterozygous alleles as missing values. MapManager program was used for constructing linkage groups. Fourteen linkage groups were generated spanning *∼*399 cM with minimum two markers for linkage group and with an LOD score threshold of 4. These linkage groups and polymorphic markers can be incorporated into existing genetic maps to generate an integrated genetic map for cotton.

### 3.4. Chromosomal Localization of MGAES Markers

**Figure 2:**
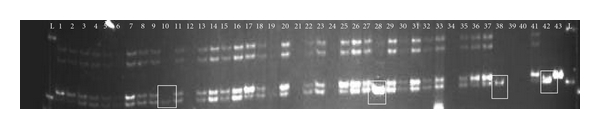
Chromosomal localization of EST-SSR marker MGAES-64 to Te11Lo (lane 10), H11 (lane 28), NTN12-11 (lane 38), and *G. barbadense* (lane 42) lines using aneuploid CS-B lines.

**Figure 3:**
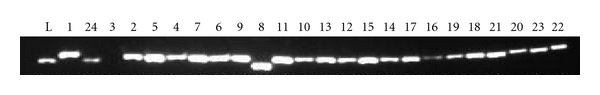
Chromosomal localization of EST-SSR marker MGAES-64 to CS-B 11Sh using euploid CS-B lines: sample 8: CS-B 11Sh, sample 24: *G. barbadense* (3–79).

Physical mapping of the polymorphic markers was facilitated using aneuploid (Figure 2) and euploid chromosome substitution lines (Figure 3). Sixteen markers were localized to different chromosomes using euploid CS-B lines while 14 markers were localized using aneuploid CS-B lines. Missing a polymorphic locus in a specific aneuploid (BC_0_F_1_) accession determines the chromosomal localization of a dominant marker to that corresponding specific chromosome or chromosome arm. Results derived from both aneuploid and euploid CS-B lines served as cross-reference to each other. For example, MGAES-64 marker was localized to H11 and Te11Lo aneuploid CS-B accessions where the accessions were deficient for chromosome 11 and its long arm, respectively; in both accessions the missing *G. hirsutum* fragment or locus has been observed confirming the localization of the marker to chromosome 11 and its long arm (Figure 2).

Using euploid chromosome substitution lines, the same MGAES-64 marker has been localized to CS-B11Sh accession, where a pair of chromosomes from *G. barbadense* was substituted for the long-arm deficient ditelosomic lines of *G. hirsutum*; polymorphic fragment corresponding to *G. barbadense* was only observed in CS-B11Sh explaining its localization to chromosome 11 long arm (Figure 3). Polymorphic markers, linkage group information, euploid, and aneuploid CS-B chromosome localization were shown in Table 3. However, we observed incongruency in localizing some markers to just a single chromosome using euploid and aneuploid CS-B analysis. This needs to be further investigated as it might be a result of duplicated loci or genome reorganization in some lines.

Our research demonstrated the use of gene-based markers for analyzing the genetic diversity among cultivated and wild cotton species. CAP markers proved more useful for detection of polymorphism in monomorphic fragments of closely related species. MGAES markers derived from *G. arboreum* ESTs were highly polymorphic and informative for developing genetic maps and other applications. Incorporation of the linkage groups and polymorphic markers into existing genetic maps help in developing integrated cotton genetic maps to assist cotton breeders.

## Supplementary Material

Supplementary File-1 contains information related to candidate gene-based markers, their primer sequences, and associated putative function. Similarly, Supplementary File-2 contains Mississippi G. arboreum EST-SSR (MGAES) primers, their source EST GenBank IDs, primer sequences, melting temperature, PCR annealing conditions, and their putative function.

## Figures and Tables

**Table 1:** Cotton genotypes used for polymorphism detection and phylogenetic tree.

No.	Species/genotype	Genome
1	*G. hirsutum/TM-1*	AD1 (CMD1)
2	*G. barbadense/3–79*	AD2 (CMD2)
3	*Acala maxxa*	AD1 (CMD3)
4	*DPL 458BR*	AD1 (CMD4)
5	*Paymaster 1218BR*	AD1 (CMD5)
6	*Fibermax 832*	AD1 (CMD6)
7	*Stoneville 4892 BR*	AD1 (CMD7)
8	*PIMA S-6*	AD2 (CMD8)
9	*G. arboreum*	A2 (CMD9)
10	*G. raimondii*	D5-3 (CMD10)
11	*G. tomentosum*	AD3 (CMD11)
12	*G. mustelinum*	AD4 (CMD12)
13	*G. darwinii*	AD5
14	*G. triphyllum*	B2
15	*G. sturtianum*	C1
16	*G. areysianum*	E3
17	*G. australe*	C3
18	*G. costulatum*	C5
19	*G. pulchellum*	C8
20	*G. thurberi*	D1
21	*G. armourianum*	D2-1
22	*G. harknessii*	D2-2
23	*G. klotzschianum*	D3K
24	*G. aridium*	D4
25	*G. gossypioides*	D6
26	*G. lobatum*	D7
27	*G. trilobum*	D8
28	*G. turneri*	D10
29	*G. schwendimanii*	D11
30	*G. stocksii*	E1
31	*G. somalense*	E2
32	*G. bickii*	G1

**Table 2:** Polymorphism information content and chromosomal localization of gene-based markers by CAP technique.

Marker	Putative gene	PIC value	Restriction enzyme	Chromosome
BG2926	Actin gene	0.949	—	—
BG7042	S-adenosyl-L-methionine decarboxylase proenzyme	0.997	—	—
BG7067	Low MW heat shock protein gene (Gmhsp17.6-L)	0.988	—	—
BG7092	Glyceraldehyde-3-phosphate dehydrogenase	0.981	—	—
BG7164	Mitogen-activated protein kinase (MAPK)	0.917	—	—
BG7197	Auxin induced basic helix-loop-helix transcription factor	0.934	—	—
BG7211	DNA-binding protein (WRKY 1)	0.967	*Rsa*I	11 short arm
BG7213	Zinc finger protein (TIM9)	0.908	—	—
BG7215	Acyl CoA independent ceramide	0.912	*Rsa*I	6
BG7226	Potassium transporter HAK3p	0.967	—	—
BG7238	Photolyase/blue light photoreceptor phr2	0.849	—	—
BG7314	Copalyl diphosphate synthase 1	0.954	*Hae*III	14 short arm, 25
BG7356	Omega-3 fatty acid desaturase (FAD3)	0.902	—	—
BG7405	Transcription factor (Hap5a)	0.967	*Hha*I	10, 16, 22 Short arm
BG7411	Ubiquitin extension protein	0.849	—	—
BG7428	Cinnamic acid 4-hydroxylase	0.794	—	—
BG7443	Small heat stress protein	0.952	*Msp*I	16
BG7446	G-protein beta subunit	0.84	—	—
BG7485	Flavonoid 3′-hydroxylase	0.998	*Hha*I	16

**Table 3:** Genetic and physical mapping of *G. arboreum*-based EST-SSR markers. (—: physical mapping was inconclusive; X: unlinked marker).

Marker	Annealing temperature (°C)	Euploid CS-B chromosome localization	Aneuploid CS-B chromosome localization	Linkage group
MGAES-2	55	—	—	LG 14
MGAES-3	55	CS-B 01	Te7Sh	LG 14
MGAES-5	55	—	—	X
MGAES-10	50	CS-B 15Sh	—	X
MGAES-11	50	—	Te9Lo, H9	LG 9
MGAES-21	55	XX	Te20Lo, H20	X
MGAES-22	55	CS-B 01	—	X
MGAES-25	55	—	—	LG 5
MGAES-27	50	—	—	X
MGAES-28	55	CS-B 10	H3Sub, Te11Lo, H11, NTN12-11	LG 7
MGAES-40	50	CS-B26Lo		X
MGAES-41	55	—	—	X
MGAES-43	50	—	—	X
MGAES-44	55	—	—	LG 5
MGAES-49	55	—	—	LG 6
MGAES-51	55	—	—	LG 6
MGAES-57	55	NTN17-11	H3Sub	X
MGAES-58	55	—	H3Sub	LG 10
MGAES-64	55	CS-B 11Sh	Te11Lo, H11, NTN12-11	LG 7
MGAES-72	55	—	—	X
MGAES-73	55	—	—	X
MGAES-78	55	CS-B 11Sh	Te11Lo, H11	LG 7
MGAES-80	55	—	—	X
MGAES-81	50	—	—	X
MGAES-82	55	—	—	X
MGAES-83	55	CS-B04, CS-B 14Sh, NTN4-15, NTN10-19	—	X
MGAES-87	55	CS-B 11Sh	Te11Lo, H10	LG 4
MGAES-91	55	—	—	X
MGAES-95	55	CS-B 02	Te2Lo, H3SuB	X
MGAES-104	50	—	—	X
MGAES-105	55	—	Te15, Te20Lo, H3Sub	LG 5
MGAES-106	55	CS-B26Lo	H12, NTN12-11	LG 3
MGAES-107	55	—	Te7Lo	X
MGAES-111	50	—	—	X
MGAES-122	50	CS-B 11Sh, NTN17-11	—	LG 12
MGAES-126	50	—	—	LG 8
MGAES-130	55	NTN4-15	H3Sub	LG 13
MGAES-135	55	CS-B 18	Te18Lo, H18	LG 1
MGAES-141	55	—	—	LG 2
MGAES-142	55	—	H10	X
MGAES-143	55	—	—	LG 11
MGAES-153	50	CS-B 01	—	X
MGAES-157	55	—	—	X
MGAES-160	50	—	—	X
MGAES-161	50	—	—	X
MGAES-165	50	—	—	X
MGAES-194	55	—	—	LG 2
MGAES-200	50	—	—	X

## Abbreviations

CAP: Cleaved amplified fragment polymorphism
EST: Expressed sequence tags
SSR: Simple sequence repeats
CS-B: Chromosome substitution lines of *G. barbadense* in *G. hirsutum *
RILs: Recombinant inbred lines
RFLP: Restriction fragment length polymorphism
AFLP: Amplified fragment length polymorphism
SNP: Single nucleotide polymorphism
CMD: Cotton Marker Database.
